# A Suggested Reform of the Lunacy Laws

**Published:** 1908-01-25

**Authors:** 


### Mental Diseases.

### A SUGGESTED REFORM OF THE LUNACY LAWS.

We have received a letter, too long for publication, complaining of the undue detention in an asylum of a relative of the writer. The patient in question has, it appears, been the subject of an Inquisition in Lunacy, has been found insane, and placed under care in a registered hospital. Our correspondent complains that she is unable to obtain the liberation of her relative, and that she has no legal status upon which she can act in the matter. She points out that when a voluntary boarder in a registered hospital becomes insane, the medical officer of the institution is bound by law to draw attention to the case and to have the patient certified, and characterises it as "an amazing anomaly" that asylum doctors should be compelled to detain patients at the will of their relatives, after they (the doctors) consider that they have effected a cure. She thinks that on the recovery of a patient the doctor should have power to procure the discharge of the patient, in spite of the opposition of the patient's relatives.

The lady is aware that the law provides the patient herself with the machinery for procuring her own release, but urges that many patients do not know what steps to take; and that even if they do act, the question is decided over the heads of the resident doctors by the intervention of a medical witness from outside, who could be influenced, and perhaps grossly misled, by the reports and representations of the opposing committee.

There are many questions raised in the letter, the chief of which we take to be these:—

1. Ought not any relative of a patient to have a legal right to initiate proceedings to procure the release of the patient?

2. Ought not the doctor in whose charge the patient is to have similar power?

3. Even if the doctor in charge of the patient has no power to initiate proceedings for the liberation of the patient, ought not his opinion to be taken as to the fitness of the patient for discharge?

Let us dispose of the third question first. Of course the doctor in actual charge of the patient is a person well qualified to give an opinion as to the fitness of the patient for discharge, and his opinion ought always to be asked. We may inform our correspondent that his opinion always is asked and duly considered. If his opinion is not sought by the person applying for the *supersedeas*, it is because that person has good reason to believe that the opinion would be adverse to the granting of that *supersedeas*.

"The subject of the lady's solicitude is a patient so found by inquisition." That is to say, she is a patient not detained on a magistrate's order made on the certificates of two doctors in the ordinary course, but a patient whose case has been formally submitted to the decision of a court of law, and who has been proved, to the satisfaction of a jury, or of a judge or master sitting without a jury, to be incapable of managing herself and her affairs: she had liberty and discretion to demand a jury at her trial if she chose to do so. The Court has appointed a committee to manage her person and another committee to manage her estate, both being bound to act under the jurisdiction of the Court, and to report and account to it for all they do. It would be difficult to imagine any safeguard more complete against improper detention.

The decision of the Court, however, is not final. It is open to the patient, after a certain lapse of time, to apply to the Court for a "*supersedeas*"—that is to say, for an order setting aside its previous decision, discharging one or both of the committees, and setting the patient at liberty. One of the complaints is that this application for a *supersedeas* cannot be made by any relative of the patient, or by the doctor in charge of the patient, but only by the patient herself or her committee. The complaint seems to us unreasonable. If the patient has not sufficient intelligence or sufficient desire for liberty to make her own application, the very strong inference is that she is still incapable of managing her affairs. The necessary procedure is simple, but it is not at all necessary for the patient to understand it, or to conduct it herself. All she need do is to employ a solicitor, and at the trial of the inquisition she must either have employed a solicitor, or have conducted her case herself. In either case she must already be quite familiar with the step it is necessary to take in order to apply for a *supersedeas*, and if she is incompetent to manage her affairs in such a matter as this, she is incompetent generally, and unfit to be entrusted with their general management.

To throw the onus of making the application upon the doctor in charge of the patient would be chimerical. Why should a man incur the expense and trouble and responsibility and anxiety of litigation on behalf of a person who is not sufficiently interested in his own affairs to undertake the litigation himself? A more absurd project has seldom been proposed, even for the reform of the lunacy laws.

So far we are taking for granted that the doctor in charge of the patient is of opinion that his patient is recovered. There is, however, no evidence of that opinion, and *primâ facie* it is incredible that he should hold it. If he thought his patient well enough to be discharged, what is to prevent him from saying "Mrs. Blank, I think you are now recovered; and if you will apply for your *supersedeas* I will support it"? Such an application, so supported, would be certain to succeed. It would be made by the party interested in making it, at her own expense and on her responsibility.

As far as we can gather from the letter, the writer is aggrieved because she has no power to institute litigation, which would burden her relative with expense for the attainment of an object which her relative does not desire, and would not benefit by.

We are always glad to open the columns of The Hospital to discussions on the lunacy laws, and suggestions for their improvement. Such discussions are not unprofitable even when, as in the present case, they show the complaints to be without foundation, and the suggestions impracticable.

